# Developing a digital phenotype to subdivide adult immunosuppressed COVID-19 outcomes within the English Primary Care Sentinel Network

**DOI:** 10.3389/fimmu.2024.1491565

**Published:** 2024-12-04

**Authors:** Meredith Leston, Debasish Kar, Anna Forbes, Gavin Jamie, Rashmi Wimalaratna, Gunjan Jiwani, José M. Ordóñez-Mena, Daniel E. Stewart, Heather Whitaker, Mark Joy, Lennard Y. W. Lee, F. D. Richard Hobbs, Simon de Lusignan

**Affiliations:** ^1^ Nuffield Department of Primary Care Health Sciences, University of Oxford, Oxford, United Kingdom; ^2^ Immunisation and Vaccine Preventable Diseases Division, UK Health Security Agency, London, United Kingdom; ^3^ UK Field Epidemiology Training Programme (UK-FETP), UK Health Security Agency, London, United Kingdom; ^4^ Department of Oncology, University of Oxford, Oxford, United Kingdom

**Keywords:** digital health, vaccine, immunosuppressed, disease surveillance, surveillance, CMR

## Abstract

**Background:**

Adults classified as immunosuppressed have been disproportionately affected by the COVID-19 pandemic. Compared to the immunocompetent, certain patients are at increased risk of suboptimal vaccine response and adverse health outcomes if infected. However, there has been insufficient work to pinpoint where these risks concentrate within the immunosuppressed spectrum; surveillance efforts typically treat the immunosuppressed as a single entity, leading to wide confidence intervals. A clinically meaningful and computerised medical record (CMR) compatible method to subdivide immunosuppressed COVID-19 data is urgently needed.

**Methods:**

We conducted a rapid scoping review into COVID-19 mortality across UK immunosuppressed categories to assess if differential mortality risk was a viable means of subdivision. We converted the risk hierarchy that surfaced into a pilot digital phenotype—a valueset and series of ontological rules ready to extract immunosuppressed patients from CMR data and stratify outcomes of interest in COVID-19 surveillance dataflows.

**Results:**

The rapid scoping review returned COVID-19 mortality data for all immunosuppressed subgroups assessed and revealed significant heterogeneity across the spectrum. There was a clear distinction between heightened COVID-19 mortality in haematological malignancy and transplant patients and mortality that approached the immunocompetent baseline amongst cancer therapy recipients, autoimmune patients, and those with HIV. This process, complemented by expert clinical input, informed the curation of the five-part digital phenotype now ready for testing in real-world data; its ontological rules will enable mutually exclusive, hierarchical extraction with nuanced time and treatment conditions. Unique categorisations have been introduced, including ‘Bone Marrow Compromised’ and those dedicated to differentiating prescriptions related and unrelated to cancer. Codification was supported by existing reference sets of medical codes; absent or redundant codes had to be resolved manually.

**Discussion:**

Although this work is in its earliest phases, the development process we report has been highly informative. Systematic review, clinical consensus building, and implementation studies will test the validity of our results and address criticisms of the rapid scoping exercise they are predicated on.

**Conclusion:**

Comprehensive testing for COVID-19 has differentiated mortality risks across the immunosuppressed spectrum. This risk hierarchy has been codified into a digital phenotype for differentiated COVID-19 surveillance; this marks a step towards the needs-based management of these patients that is urgently required.

## Introduction

Despite it being well-known that the immunosuppressed population experience worse respiratory infection outcomes, large administrative or surveillance datasets rarely collect data on how these might vary between condition types (1–4). Reports are either aggregated, handling immunosuppressed data as a singular entity (5), or only return differentiated data for dominant subgroups, such as cancers (6). This erases wider sub-trends that may be of clinical interest and undermines evidence-based prioritisation of medical resources including vaccines and their passive alternatives (7).

The Oxford-Royal College of General Practitioners (RCGP) Research and Surveillance Centre (RSC) is well-positioned to respond to recent appeals (8) made by its partners, the United Kingdom Health and Security Agency (UKHSA) to improve the granularity of immunosuppressed COVID-19 surveillance. The trusted research environment it utilises, the Oxford-RCGP Clinical Informatics Digital Hub (ORCHID) (9), offers nationally representative (n > 20 million) and contemporaneous data from computerised medical records (CMR) and biological specimen data – ideal for differentiated work in complex populations or remits (10). This paper documents our first steps to enhancing RCGP RSC surveillance of immunosuppressed patients by delivering an ORCHID-compatible and COVID-19 specific digital phenotype for ‘Adult immunosuppression’ – a phenotype being a set of rules that can be applied to CMR system data to identify or differentiate specific cohorts and/or episodes of interest (11). In the absence of a gold standard (5), we carried out a three-step process to deliver a testable pilot of this digital tool.

Leveraging international testing campaigns and their resultant research outputs (12), we first assessed whether COVID-19 mortality data was retrievable for the entire immunosuppressed spectrum and, if so, whether visible clusters of risk existed to inform hierarchical stratification. We then utilised in-house clinical expertise to convert this conceptual hierarchy into a valueset compatible with CMR systems. Finally, in-house data expertise developed the logistical rules that could express this hierarchy within a data extract, allocating medical records into risk levels in accordance with the underpinning hierarchy while respecting key time and treatment dependencies and the need for mutual exclusivity. The present paper describes the outcomes of these exercises and the challenges and key learnings that emerged.

This work was conducted in the UK context, where *Immunisation against infectious disease* (Green Book) chapters set-out national vaccine policy and offer the textual phenotype for ‘Adult Immunosuppression’ used in this remit (13). However, unlike international analogues (14), Green Book does not supply detail on differential risk or stratify its characterisations of immunosuppression in any way. To the best of the authors’ knowledge, this work is therefore the first to attempt to convert this specific resource into a format compatible with a hierarchical database search of the immunosuppressed and their COVID-19 outcomes. By doing so, it is our intention to create a feedback loop of real-world evidence to continually refine, augment and target Green Book COVID-19 guidelines for the benefit of immunosuppressed patients and the clinicians that treat them.

## Method

### Rapid scoping review

The rapid scoping review was performed as a hypothesis generating exercise. It was requested by stakeholders at the United Kingdom Health Security Agency (UKHSA) to establish whether COVID-associated mortality could be extracted and compared across all immunosuppressed conditions and medications listed within *Immunisation against infectious disease* (Green Book) Chapter 14a (**Box 1**) (13) and if clusters of risk were visible at surface-level review. This request was made during the second year of the pandemic and leveraged the scale and specificity of the immunosuppressed COVID-19 literature base that had accumulated even by that early stage thanks to the proliferation of community testing (12). When attempted, there was insufficient data to complete this exercise for immunosuppressed Seasonal Influenza outcomes (**Supplementary Materials**).

Box 1 **Immunosuppression in adult populations as defined by *Immunisation against infectious disease* (Green Book) Chapter 14a**

Immunosuppression

Immunosuppression due to disease or treatment, including patients undergoing chemotherapy leading to immunosuppression, patients undergoing radical radiotherapy, solid organ transplant recipients, bone marrow or stem cell transplant recipients, HIV infection at all stages, multiple myeloma or genetic disorders affecting the immune system (e.g. IRAK-4, NEMO, complement disorder, SCID).

Individuals who are receiving immunosuppressive or immunomodulating biological therapy including, but not limited to, anti-TNF, alemtuzumab, ofatumumab, rituximab, patients receiving protein kinase inhibitors or PARP inhibitors, and individuals treated with steroid-sparing agents such as cyclophosphamide and mycophenolate mofetil.

Individuals treated with or likely to be treated with systemic steroids for more than a month at a dose equivalent to prednisolone at 20mg or more per day for adults.

Anyone with a history of haematological malignancy, including leukaemia, lymphoma, and myeloma.

Those who require long-term immunosuppressive treatment for conditions including, but not limited to, systemic lupus erythematosus, rheumatoid arthritis, inflammatory bowel disease, scleroderma, and psoriasis.

This exercise applied the following PECO structure: Population – COVID-19 infection; Exposure – Adult Immunosuppression (as defined by Green Book Chapter 14a (13)); Comparator – Immunocompetent; Outcome – COVID-19 mortality. As summarised in **Box 2**, all medications, procedures and conditions listed in Green Book Chapter 14a were entered into Google Scholar as search terms along with mortality- and COVID-19-related language. Overlapping or duplicated terms were collapsed into common categories for simplicity and specific inclusion and exclusion criteria were applied. Searches were iterative and utilised the ‘relevant articles’ function of Google Scholar to redundancy. Search dates were January, 2020 to August, 2022 inclusive.

Box 2 **Example Search Terms and Inclusion & Exclusion Criteria for Rapid Literature Review**

Search terms included in Google Scholar search:

COVID-19 AND “excess mortality” AND chemotherapy OR radiotherapy OR “solid organ transplant*” OR transplant OR “bone marrow” OR “stem cell” OR myeloma OR complement OR biologics OR “biological therap*” OR immunotherapy OR PID OR HIV OR AIDS OR steroid OR prednisolone OR malignancy OR lymphoma OR “rheumatoid arthri*” OR IBS OR scleroderma OR lupus OR psoriasis OR “steroid sparing” OR “anti-TNF” OR leukaemia OR “genetic disorder*” OR rituximab OR “protein kinase inhib*” OR “PARP*” OR cyclophosphamide OR “mycophenolate mofetil” OR “long term immuno*” OR “long-term immuno*” OR IRAK-4 OR NEMO OR SCID OR alemtuzumab OR ofatumumab.

Inclusion criteria:

• Study investigated the impact of COVID-19 on a specific immunosuppressed category (as defined by *Immunisation against infectious disease* [Green Book] Chapter 14a).

• Mortality data were provided in some form (proportion deceased and/or comparative mortality to immunocompetent via effect measure).

• Study reported adult data (over 18 years).

Exclusion criteria:

• Study did not investigate the impact of COVID-19 on a specific immunosuppressed category (as defined by *Immunisation against infectious disease* [Green Book] Chapter 14a).

• Mortality data were not provided in any form.

• Multiple/overlapping risk groups were assessed (e.g. pregnant/diabetic immunosuppressed patients).

• Immunosuppressives were investigated as a prospective treatment for COVID-19, not as a risk factor.

• Study included paediatric data (under 18 years).

Exemplar studies on the COVID infection experience of each immunosuppressed category were selected - a selection process that considered sample size, research design, data completeness and efforts to minimise bias. Screening for exemplar studies occurred first by title and abstract before full text was assessed and data was extracted where relevant. This was also an iterative process, with exchanges to certain exemplar studies made when invalid or superior data was identified upon second review. An extraction form (populated further within) supported the collection of all necessary exemplar study characteristics, including Green Book category, Title, Author(s), Year, Country, Excess COVID Mortality (Effect Measure), 95% Confidence Interval, Proportion Mortality amongst All Case Types and Proportion Mortality in Hospitalised Cases. Quality assured by expert clinicians on an ongoing basis, observed clusters of effect sizes (denoting excess mortality between immunosuppressed categories and immunocompetent counterparts) and mortality percentages informed our conceptual means of subdivision.

### Developing the phenotype

**Figure 1:**
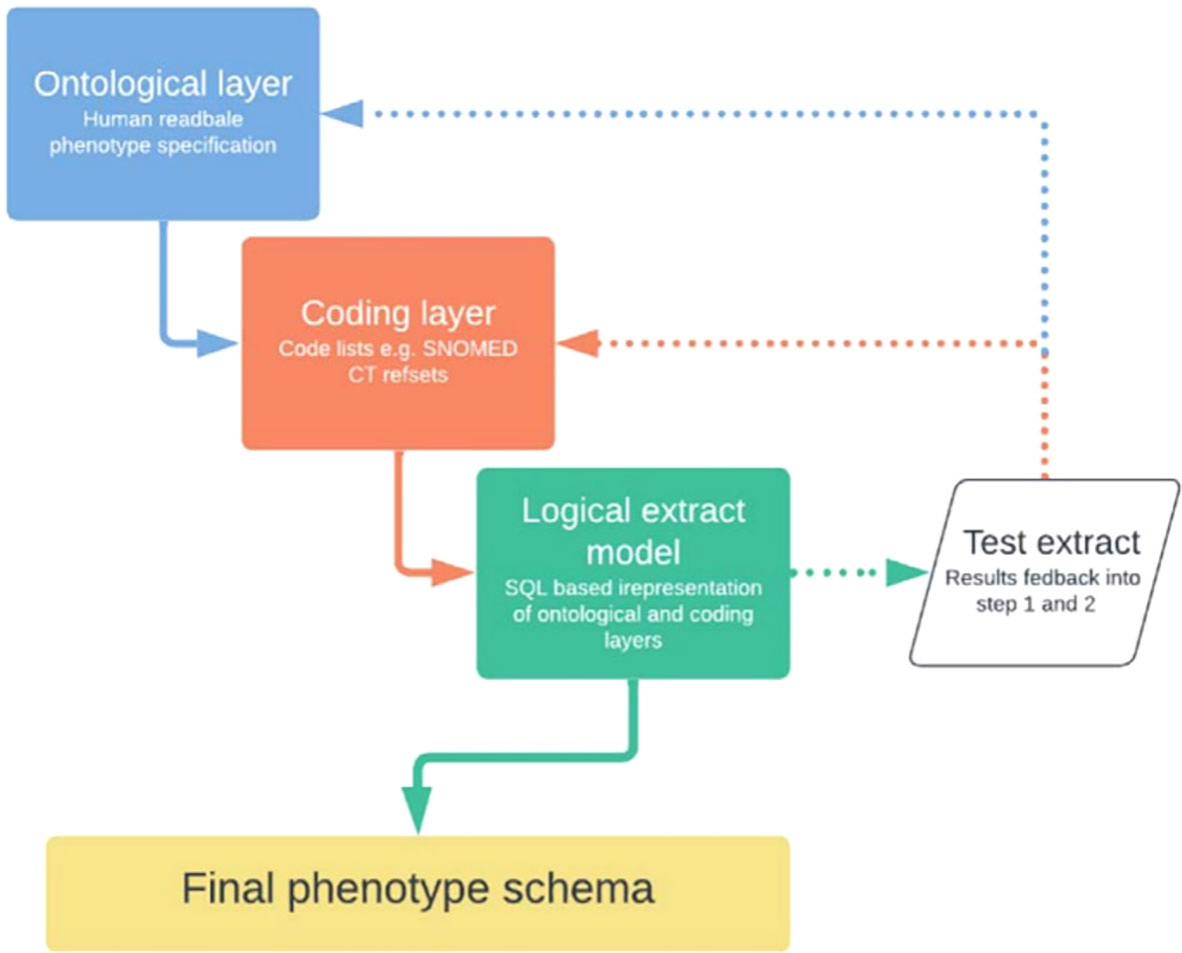
Three-layered approach to instituting a novel phenotype in RSC data.

As illustrated within **Figure 1** (15), the RSC currently utilises a three-layered approach to preparing a novel phenotype for use within its data flows:

1. An ontological layer - a human-readable description of key concepts within, and relationships between, a given phenotype.
2. A coding layer – where conceptual phenotype layers are translated into their constituent clinical codes and thematic categories.
3. A logical data extract layer – where ontological rules, expressing the relationships between phenotype levels and their respective eligibility criteria, are converted into Structured Query Language (SQL) expressions to extract desired data from the overall pool(s) available.

This process was applied when converting the conceptual framework of immunosuppression that surfaced via rapid scoping review (our ontological layer) into a test-ready phenotype (test extract). The final phenotype schema will only be confirmed and published once the pilot phenotype has been tested and validated within the ORCHID. To be compatible with this server, the curation discussed here only utilised Systematized Nomenclature of Medicine Clinical Terms (SNOMED CT) diagnostic and Dictionary of Medicines and Devices (dm+d) prescription terms.

UKHSA currently commissions the University of Nottingham based Primary Care Information Services (PRIMIS) (16) to translate its risk groups into a SNOMED CT and dm+d reference set for its disease surveillance needs, including immunosuppression. PRIMIS versions 1.3.3 and 2.3 were consequently a key resource for our coding layer. However, PRIMIS is undifferentiated and is sensitive to redundancy as SNOMED CT and dm+d vocabularies are updated on a regular basis (monthly and weekly, respectively). Furthermore, PRIMIS clinical risk groups can be highly porous, with codes relevant for immunosuppression located in numerous other categories.

As such, to create our valueset, all immunosuppressed PRIMIS terms had to be manually extracted, updated or scraped from wider risk groups using an in-house SNOMED CT Concept ID tool (described elsewhere (17)). Supertype and theme variables specific to the ORCHID TRE, known as Themes Access Dynamic Data Services [TADDS] variables, were also included in our coding layer to improve the comprehensiveness of our valueset and to reduce its vulnerability to redundancy [ibid]. Each with their own ID, TADDS variables denote specific disease or medication types and can be easily updated using Expression Constraint Language (ECL). Once completed, this valueset could then be subdivided in accordance with rapid scoping review findings; this final immunosuppressed valueset (provided in **Supplementary Materials**) was then cross-checked for accuracy and comprehensiveness against Chapter 14a specifications. Multiple expert clinicians (DK, AF, RW, GJ) quality assured this process.

The logical extract model aimed to incorporate the time conditions specified in Chapter 14a immunosuppressed criteria (13) and relevant literature (18) into our pilot phenotype. Furthermore, hierarchical principles were applied to ensure that, once tested within real-world data, our pilot phenotype would eliminate multi-level allocation of medical records. This mutual exclusivity ensured patients would only be allocated into, and contribute their data to, the highest risk level they were eligible for.

## Results

### Rapid scoping review

Due to the hypothesis-generating nature of this work, its iterative nature and the time-sensitivity involved, data was not retained on the number of articles returned, reviewed or excluded.

**Figure 2:**
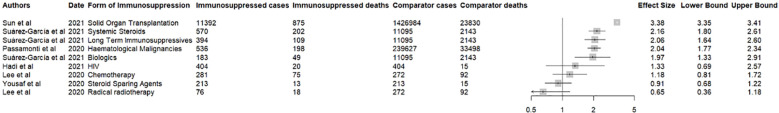
Excess COVID-19 mortality data between Green Book immunosuppressed categories and immunocompetent comparators.

Once overlapping or synonymous immunosuppressed categories were collapsed, twelve categories were left to be populated with their respective exemplar cohort studies (19–27). The data extraction form that supported this process is provided in **Table 1** below. Exemplar studies that met search criteria, reported excess mortality between immunosuppressed subgroups and their immunocompetent comparators are reported in **Figure 2**. Here, ‘Effect Size’ reflects the variety of measures that were extracted, including Odds, Hazard and Risk Ratios. The mathematical equivalence of these measures, as argued by Symons & Moore (2002) (28), justified our decision to report unadjusted data via this umbrella term.

**Table 1:** Completed data extraction form of exemplar rapid scoping review cohort studies comparing COVID-19 associated mortality between immunosuppressed categories and immunocompetent comparators.

Green Book Category	Reference	Year	Country	Immunosuppressed cases (n)	Immunosuppressed mortality (n)	Comparator cases (n)	Comparator mortality (n)	Effect Size	LCI	UCI	All case mortality* (%)	Hospitalised mortality** (%)
Chemotherapy	Lee et al. (19)	2020	UK	281	75	272	92	1.18	(0.81	1.72)	Not reported	27.0%
Radical radiotherapy	Lee et al. (19)	2020	UK	76	18	272	92	0.65	(0.36	1.18)	Not reported	24.0%
Solid Organ Transplantation	Sun et al. (20)	2021	USA	11392	875	1426984	23830	3.38	(3.35	3.41)	7.68%	Not reported
Bone Marrow/Stem Cell Transplantation	Ljungman et al. (21)	2021	Global	382	95	Not reported	Not reported	Not reported	Not reported	Not reported	25.20%	Not reported
HIV	Hadi et al	2021	USA	404	20	404	15	1.33	(0.69	2.57)	4.95%	Not reported
Multiple Myeloma	Chari et al. (23)	2020	Global	617	203	Not reported	Not reported	Not reported	Not reported	Not reported	Not reported	33.0%
Primary Immunodeficiencies	Shields et al. (24)	2021	UK	310	55	Not reported	Not reported	Not reported	Not reported	Not reported	17.70%	Not reported
Biologics	Suárez-García et al. (25)	2021	Spain	183	49	11095	2143	1.97	(1.33	2.91)	Not reported	26.7%
Steroid sparing agents	Yousaf et al	2020	Global	213	13	213	15	0.91	(0.68	1.22)	6.10%	Not reported
Systemic steroids	Suárez-García et al. (25)	2021	Spain	570	202	11095	2143	2.16	(1.8	2.61)	Not reported	35.4%
Haematological malignancies	Passamonti et al. (27)	2020	Italy	536	198	239627	33498	2.04	(1.77	2.34)	37.00%	Not reported
Long Term Immunosuppressives	Suárez-García et al. (25)	2021	Spain	394	109	11095	2143	2.06	(1.64	2.6)	Not reported	27.7%

* Percentage of deaths recorded amongst all COVID cases.

** Percentage of deaths recorded exclusively amongst cases hospitalised with COVID.

Exemplar study estimates of COVID-19 mortality risk varied considerably across the immunosuppressed population. Higher versus lower risk categories are identifiable, with agreement between mortality percentages and effect size estimates on the elevated vulnerability of haematological malignancy patients and recipients of systemic steroids and of minimal excess mortality seen amongst HIV, chemotherapy and radiotherapy patients. Disparities in risk can also be seen within immunosuppressed condition and medication types (solid tumours versus haematological malignancies and biologics versus cancer therapies, respectively). There were notable discrepancies between the risk profiling that emerged from mortality percentages versus effect size estimates, however. The elevated effect size of solid organ transplant recipients is not borne out in percentage data, for example.

Due to the heterogeneity of exemplar studies’ designs and sample sizes, effect measure-based estimates of COVID-19 mortality were prioritised over percentage data. This comparative metric standardised between-category estimates and made for fairer comparisons than would be achieved using absolute values.

The following three patient clusters proved most valuable for dividing immunosuppressed COVID-19 data in the first instance: 1) those with bone marrow compromising conditions, 2) those receiving ongoing immunosuppressing treatments and/or procedures and 3) those with primary or acquired immunodeficiencies. To prevent oversimplification, RSC clinicians (DK, AF) expanded upon this initial tripartite model to create the five-part phenotype elaborated upon in the next section of reporting. Here, dedicated strata were created for solid organ transplant recipients and immunosuppressive regimens related and unrelated to cancer in recognition of their unique risk profile and easy confusion in large databases respectively (the same immunosuppressive agents can be prescribed as cancer and non-cancer therapies). **Table 2** provides brief descriptions of these five levels and their respective immunosuppressive conditions or treatment regimens.

**Table 2:** Composition of Pilot Phenotype for ‘Adult Immunosuppression’, stratified by COVID-19 mortality risk.

Phenotype Level	Composition
1. Bone Marrow Compromising Conditions	Haematological malignancy and Bone Marrow/Stem Cell Transplantation
2. Solid Organ Transplant Recipients	Solid Organ Transplantation
3. Non-Cancer Immunosuppressive Treatments	Immunosuppressives unrelated to cancer
4. Recent Cancer Treatments	Systemic cancer treatments including cancer-related immunotherapy, chemotherapy and radical radiotherapy
5. Immunodeficiency	Genetic immunodeficiencies and HIV/AIDS

### Developing the phenotype

**Figure 3:**
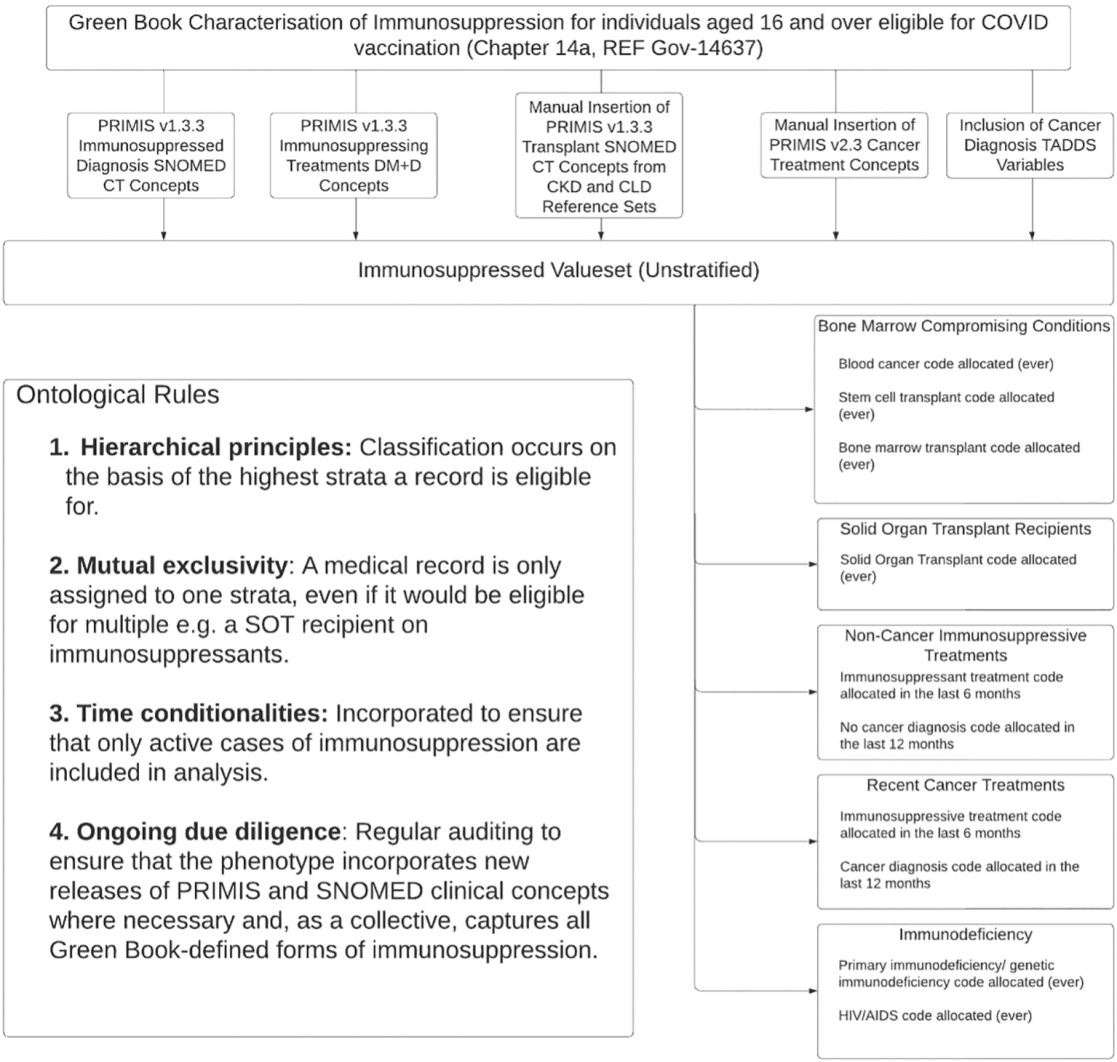
Clinical logic flow of the immunosuppressed phenotype.

The test valueset is provided in **Supplementary Materials**. As illustrated within **Figure 3**, a combination of PRIMIS terms and cancer-specific TADDS IDs translated our conceptual five-part phenotype into this SNOMED and dm+d compatible format. Due to a major SNOMED CT update over the course of the curation process, new terms for newly redundant or retired codes had to be identified and manually inserted. As part of this process, PRIMIS sources also went through additional cross-checks for immunosuppression-eligible codes located in broader risk categories; several transplantation codes were scraped from chronic kidney and chronic liver disease categories, for example. Finally, the cancer diagnosis TADDS variables that differentiated between Non-Cancer Immunosuppressive Treatments and Recent Cancer Treatment layers were curated and quality assured by colleagues within the Nuffield Department of Primary Care Health Sciences, including RSC clinical and data team members (DK, GJ).

Operationalised with SQL, ontological rules for hierarchical mutual exclusivity, time sensitivity and diagnosis-treatment pairing stratify this valueset into our desired pilot phenotype for ‘Adult Immunosuppression’ for onward use in COVID-19 surveillance. Rules currently follow Green Book time directives (13) and best practice from contemporary literature (18) to exclude those that would be considered insufficiently immunosuppressed during extraction (e.g. legacy cancer patients and those whose immunosuppressive regimen discontinued greater than 6 months prior to infection date). This pilot phenotype is now being tested within ORCHID data; results will be reported imminently. Any difficulties, inaccuracies or misclassifications that surface during this test will be used to refine the phenotype prior to its publication as our final phenotype schema within BioPortal (29), PhenoFlow (30) or an equivalent.

## Discussion

This paper outlines the development of a risk-stratified, test-ready phenotype for ‘Adult Immunosuppression’ for COVID-19 surveillance dataflows. To the best of our knowledge, this work is the only to operationalise UK specifications for immunosuppression in a hierarchical manner. Utilising COVID-19 mortality as our means of subdivision, this pilot phenotype represents a novel, clinically relevant and medical health record compatible basis for identifying the immunosuppressed in CMR repositories and appraising their COVID-19 outcomes by vulnerability level. Once implemented, this has significant prospective value for COVID-19 surveillance and, if validated, patient triage and treatment prioritisation. Due to the idiosyncrasies of COVID-19 (31), transferability of this phenotype to other disease domains can only be speculated at this stage, however.

Our pilot phenotype has already benefitted from the oversight and quality assurance of the in-house clinical team of the RSC. Work is now underway to assess its capabilities in real-world data; feasibility exercises within ORCHID will be published in due course. As per Methods, findings will be used to either validate or feedback and refine the pilot phenotype.

Despite the prospective value of this pilot phenotype, it must be restated that this work was intended as a hypothesis generating exercise; its explorative and rapid nature makes it inappropriate for general use and the valueset provided requires further validation before we can recommend this.

In the first instance, risk stratification was based on single-study estimates for COVID-19 mortality and the practical requirements of CMR extraction. The superficiality of the rapid scoping exercise that procured this data warrants criticism – its transparency and replicability were both undermined by failing to pre-publish a protocol and retain precise screening data. Furthermore, the heterogeneity of exemplar studies’ designs and sample sizes confounded comparisons between absolute mortality outcomes. While effect measure data (denoting excess mortality as compared to immunocompetent counterparts) could compensate by providing a more standardised form of comparison, they were not reported for all subgroups. A systematic replication and meta-analysis were recently conducted (32) to address all these issues and to test the rigour of pilot phenotype levels. Despite the general concordance between the two exercises, the results of this systematic work suggest some inaccuracies in our pilot phenotype – most notably the collapsing of acquired with primary immunodeficiencies given the generally elevated risk profile of the latter (33) – and, thanks to its global scope, some key geographical distinctions. COVID infection carries very different risks for the HIV patient with access to antiretroviral therapy versus without, for example (34). An eDelphi study is currently underway to leverage this evidence to produce a more definitive COVID-19 risk stratified phenotype for ‘Adult Immunosuppression’ (35).

The expression of our pilot phenotype is also not without its limitations. In the first instance, the mutual exclusivity that was codified between phenotype levels does not exist in reality. Immunosuppressive conditions rarely go untreated, for example. This will likely make it difficult to demarcate mortality risks between diagnoses and their medications. Furthermore, the extensional nature of the pilot phenotype, manually curating levels on a code-by-code basis, makes it susceptible to inaccuracies and redundancies as both PRIMIS and its underpinning SNOMED and dm+d clinical concepts update on a regular basis. While rigorous quality assurance efforts and intentional use of supertype terms and TADDS variables makes us confident that our valueset is both reflective of the diversity of Green Book immunosuppressed terms and somewhat resilient to redundancy, future iterations of this work would benefit from intensional – rule-based, machine-readable and more readily-sharable – curation methods. In the spirit of open science, this will be looked to as the standard for all future RSC phenotypes (17).

Finally, the implementation of the pilot phenotype may prove challenging in certain areas. Automating patient allocation via time and dual treatment-diagnosis is complex and may lead to inaccuracies when applied to computerised medical records; conditional levels, most notably those that are medication-specific, are far harder to extract than their unconditional and diagnostic-specific counterparts. For example, 12-month caps on cancer diagnostic codes and 6-month minimums for immunosuppressive medications were incorporated to prevent the inclusion of legacy cancers or low-level immunosuppression respectively. However, such crude constraints may lead to oversimplification, under sampling or misclassification in practice - especially when considering well-documented issues in keeping primary care records and prescription data updated (36). These caps are also at odds with more recent clinical logic to identify immunosuppressed patients (37); this has extended cancer treatment caps to 12 months and 24 months for chemo/radiotherapy and Chimeric Antigen Receptor T-cells (CAR-T) therapy respectively. This work also failed to operationalise Green Book dose dependencies for immunosuppression (e.g. equivalence to ≥ 20mg of prednisolone) which is a major limitation. In acknowledgment of the challenges of automating drug-inclusive phenotypes for immunosuppression, future iterations of this work may wish to consider condition-specific phenotyping or binary alternatives (higher vs lower risk) – either precluding medication codes altogether or simplifying allocation. Clinical consensus will be built on these topics via the eDelphi exercise previously mentioned (35).

## Conclusion

Despite its hypothesis-generating intentions, this pilot phenotype for ‘Adult Immunosuppression’ from a COVID-19 lens is the first we are aware of to automate the identification and risk-stratification of this ill-defined and highly vulnerable population. Its development and curation have already returned valuable insights for those working to enhance COVID-19 surveillance and its first trial in real-world data is underway. This validation is urgently needed, though concordances between the rapid scoping review described here and its systematic replication (32) are encouraging. Results of both this and the eDelphi study (35) to build international consensus on immunosuppressed definitions and COVID risks will be utilised to create and publish a final schema phenotype of ‘Adult immunosuppression’ for COVID-19 dataflows. Until this point, the materials provided in this paper should not be considered ready for general use.

## Data Availability

The original contributions presented in the study are included in the article/**Supplementary Material**. Further inquiries can be directed to the corresponding author.
